# Insights into immuno-oncology drug development landscape with focus on bone metastasis

**DOI:** 10.3389/fimmu.2023.1121878

**Published:** 2023-07-05

**Authors:** Tiina E. Kähkönen, Jussi M. Halleen, Gary MacRitchie, Ronnie M. Andersson, Jenni Bernoulli

**Affiliations:** ^1^OncoBone Ltd, Oulu, Finland; ^2^1stOncology, BioSeeker Group AB, Stockholm, Sweden; ^3^University of Turku, Institute of Biomedicine, Turku, Finland

**Keywords:** cancer, bone metastasis, immuno-oncology, osteoimmuno-oncology, immunotherapies, drug development, 1st oncology, database

## Abstract

Bone is among the main sites of metastasis in breast, prostate and other major cancers. Bone metastases remain incurable causing high mortality, severe skeletal-related effects and decreased quality of life. Despite the success of immunotherapies in oncology, no immunotherapies are approved for bone metastasis and no clear benefit has been observed with approved immunotherapies in treatment of bone metastatic disease. Therefore, it is crucial to consider unique features of tumor microenvironment in bone metastasis when developing novel therapies. The vicious cycle of bone metastasis, referring to crosstalk between tumor and bone cells that enables the tumor cells to grow in the bone microenvironment, is a well-established concept. Very recently, a novel osteoimmuno-oncology (OIO) concept was introduced to the scientific community. OIO emphasizes the significance of interactions between tumor, immune and bone cells in promoting tumor growth in bone metastasis, and it can be used to reveal the most promising targets for bone metastasis. In order to provide an insight into the current immuno-oncology drug development landscape, we used 1stOncology database, a cancer drug development resource to identify novel immunotherapies in preclinical or clinical development for breast and prostate cancer bone metastasis. Based on the database search, 24 immunotherapies were identified in preclinical or clinical development that included evaluation of effects on bone metastasis. This review provides an insight to novel immuno-oncology drug development in the context of bone metastasis. Bone metastases can be approached using different modalities, and tumor microenvironment in bone provides many potential targets for bone metastasis. Noting current increasing interest in the field of OIO, more therapeutic opportunities that primarily target bone metastasis are expected in the future.

## 1 Introduction

Metastases are the main cause of cancer-related deaths and bone is among the major sites of metastasis in many cancers such as breast, prostate, lung, renal, colon and bladder cancer and melanoma (1). Notably, when bone metastases are observed, the 5-year survival rate drops to 5% as there are no effective treatments available (2). Therefore, bone metastases are incurable and induce severe skeletal-related effects such as pathological fractures, spinal cord compression, bone pain and decreased quality of life (3).

Immunotherapies may give hope for bone metastatic patients (4). Bone marrow is an important secondary lymphoid organ and bone metastases develop a unique immune microenvironment. Bone is a highly immunosuppressed microenvironment, as recently demonstrated for prostate cancer (5), which may explain why immunotherapies have not produced promising effects on bone metastatic patients (6). Factors behind the development of the immunosuppressed bone metastatic microenvironment include: 1) in the process of metastasis formation, cancer cells need to have properties that allow them to avoid elimination by immune cells, 2) even at healthy state, bone marrow has a lower number of cytotoxic cells than other tissues, 3) immunomodulation of the pre-metastatic niche to allow seeding of cancer cells to the growth-supporting microenvironment, and 4) modulation of the metastatic microenvironment through interactions of stromal cells.

The role of stromal cells in modulating the microenvironment is often neglected. Stromal cell effects are especially important in bone metastasis because bone cells are important regulators in promoting tumor growth in a process called vicious cycle of bone metastasis (7). The vicious cycle explains how cancer cells regulate the number and activity of bone resorbing cells, which in turn results in increased bone resorption and release of factors that promote growth of bone metastases. Furthermore, stromal cells have a role in modulating immune microenvironment (8). For example, bone-resorbing osteoclasts can present antigens, inhibit T cells and express immunosuppressive factors (9), which also occurs in bone metastases (10).

A recently established novel osteoimmuno-oncology (OIO) concept refers to interactions between cancer, bone and immune cells (11). It is essential to understand these interactions in order to develop effective and safe therapies for cancer patients with bone metastases. The OIO concept is supported by years of research on the role of interactions between cancer, bone and immune cells, and also by observations in patients treated with different immunotherapies who developed skeletal-related adverse events (SRAEs) such as resorptive bone lesions, spinal cord compression and even fractures (12). A recent study indicated that many musculoskeletal adverse effects are observed in immunotherapy-treated patients, but interestingly, patients experiencing musculoskeletal adverse effects had a good anti-tumor response (13). This could be explained by changes in the immune microenvironment that make the tumors responsive to immunotherapies but at the same time disturb the immune homeostasis in bone, leading to above-described adverse events. Therefore, development of novel therapies with confirmed efficacy on bone metastasis and without causing SRAEs should be prioritized especially for bone metastatic patients who already have compromised bone health.

This review summarizes current immuno-oncology drug development landscape for bone metastatic breast and prostate cancer. Using a comprehensive oncology-focused drug development database we identified drugs with preclinical or clinical data in the context of bone metastasis. Approved immunotherapies were excluded from this review as they have recently been discussed elsewhere (4). Current treatment options for bone metastasis, all with limited efficacy, include radium-223 dichloride, bisphosphonates such as zoledronic acid, and the anti-RANKL antibody denosumab, that can be applied in combination with standard-of-care cancer therapies.

## 2 Database search for immuno-oncology drugs in development for bone metastatic breast and prostate cancer

This unique and comprehensive data review was performed using 1stOncology database that contains detailed scientific, clinical and commercial drug information on almost 20,000 oncology drugs and 1,877 targets and covers more than 21,000 interventional clinical trials in 391 indications (search results on November 18, 2022). The research concentrated on breast and prostate cancers that have the highest incidence of bone metastases (14).

The search of active studies for breast cancer resulted in 1,498 drugs and 537 targets. Addition of ‘bone metastasis’ in the search resulted in 242 drugs and 178 targets. Of these drugs, 67 were immuno-oncology drugs that are of interest to this review, of which 36 were in clinical and 1 in preclinical development. In prostate cancer, there were 746 drugs in active research, covering 356 targets. Limiting down to those associated with bone metastasis resulted in 206 drugs and 180 targets. Of these drugs, 73 were in the immuno-oncology category, of which 47 in clinical and 2 in preclinical development. These search results are summarized in **Table 1**, and these 86 drugs (36 + 1 for breast and 47 + 2 for prostate cancer) were the starting population for a more detailed search for efficacy data on bone metastasis. The more detailed search included relevant scientific results published in major scientific events. After going through all drugs identified in the initial search it was concluded that 20 drugs in clinical development had published data available about effects on bone metastasis that can be discussed in this review.

**Table 1:** Database searches performed to narrow the scope of this review.

Database search	Breast cancer	Prostate cancer
All drugs in active development	1,498 drugs	746 drugs
Limiting to drugs with development related to bone metastasis	242 drugs	206 drugs
Further Limiting to immuno-oncology drugs only	67 drugs	73 drugs
Further Limiting to immuno-oncology drugs in clinical development (phase 1-3)	36 drugs	47 drugs
**Drugs with published data available about effects on bone metastasis**	0 drugs	20 drugs*

* Of the 20 drugs, 6 included both breast and prostate cancer as indication, but bone metastasis data is published only in prostate cancer bone metastasis.

## 3 Immuno-oncology drug development for bone metastasis

### 3.1 Overview of immuno-oncology drugs in clinical development

**Table 2** lists the 20 prostate cancer therapies in clinical development with published data on bone metastasis effects. Notably, even though the initial search identified therapies also for breast cancer, none of the clinical trials in breast cancer specifically addressed effects on bone metastasis. Inclusion criteria for the trials listed bone metastases evaluated by imaging, and outcome measurements varied largely between studies. Most of the listed trials are currently in phase 1 or 2. Description of the main findings in the context of bone metastasis for all listed therapies is provided in the chapters below.

**Table 2:** Immuno-oncology drugs in clinical development for prostate cancer in the context of bone metastasis.

ASSET NAME	TARGET(S)	MODA-LITY	TRIAL	SELECTED ELIGIBILITY CRITERIA AND OUTCOME MEASUREMENT		TRIAL STATUS	DEVELOPER/SPONSOR
				INCLUSION	OUTCOME		
Imifoplatin (PT-112)	Pyrophos-phate-platinum conjugate	Small molecule	NCT02266745, ph1/2	Progressive disease measured by physical examination or imaging (RECIST v1.1 or PCWG3 or by informative tumor markers)	Secondary: rPFS, disease control rate, objective response rate, duration of response, OS	Recruiting	Promontory Therapeutics/ Pfizer, EMD Serono
P-PSMA-101	PSMA	CAR-T	NCT04249947, ph1 (in combination with rimiducid)	Measurable disease by RECIST 1.1 or bone only metastases with measurable PSA	Overall response rate, percentage of patients with complete or partial response	Recruiting	Poseida Therapeutics
Pasotuximab (BAY2010112)	PSMAxCD3	Bispecific antibody	NCT01723475, ph1	Appearance of one more new lesions in bone scan	Secondary: Tumor and PSA response	Completed	Bayer
MGC018	B7-H3	Antibody-drug conjugate	NCT03729596, ph1/2 (combination with anti-PD-1 will not enroll) NCT05551117, ph2/3	In prostate cancer cohort, patients with bone only disease are eligible One or more metastatic lesion, present MRI, CT or bone scan	Secondary: OS, PFS, rPFS, response rate Primary: rPFS	Active, not recruiting Not yet recruiting	MacroGenics
DS-7300a	B7-H3	Antibody-drug conjugate	NCT04145622, ph1/2	CRPC participants with bone only disease may be eligible on a case-by-case basis	Anti-tumor activity	Recruiting	Daiichi Sankyo
MVI-118	Encode AR LBD	DNA vaccine	NCT02411786, ph1 (+/- GM-CSF)	Soft tissue and/or bone metastases by radiographic imaging	Secondary: Median and 18-month PFS	Completed	Madison Vaccines
MVI-816 (pTVG-HP)	Encode AR LBD	DNA vaccine	NCT01706458, ph2 (in combination with sipuleucel-T) NCT02499835, ph1/2 (in combination with pembrolizumab)	Soft tissue and/or bone metastases in imaging studies	Secondary: PFS, time to radiographic progression	Completed Active, not recruiting	Madison Vaccines
Recombinant Ad5 vaccine	PSA/MUC-1/brachyury	Virus vaccine	NCT03481816, ph1	Metastatic bone disease in an imaging study	Secondary: OS, PFS	Completed	ImmunityBio/NCI
Rilimogene glafolivec	PSA, CD48, CD80, ICAM1, KLK3	Virus vaccine	NCT01322490, ph3 (+/- GM-CSF)	Radiological progression (new or growing bone metastases or new/enlarging lymph node disease)	Primary: OS, number alive without event after 6 months (event is two new bone lesions or other metastases)	Completed	Bavarian Nordic
Bintrafusp alfa (M7824) and M9241	PD-L1- TGFβ NHS- IL12A	Fusion protein Fusion protein	NCT04633252, ph1/2 (in combination with androgen deprivation therapy, prednisone and docetaxel)	Metastatic disease, defined as at least one lesion on TC99 bone scan or at least one measurable lesion per RECIST 1.1.	Secondary: Radiographic response rates, radiographic and biochemical time to progression	Recruiting	Merck KGaA/NCI
Vudalimab (XmAb20717)	PD-1 x CTLA-4	Bispecific antibody	NCT05005728, ph2 (in combination with carboplatin, cabazitaxel, olaparib	Progression of bone disease (evaluable disease) or 2 or more new bone lesions by bone scan	Secondary: Objective response rate by PCWG3, bone scan and rPFS, duration of response	Recruiting	Vencor
Tremelimumab	CTLA-4	Monoclo-nal antibody	NCT03204812, ph2 (in combination with durvalumab)	Evidence of metastatic disease to the bone seen in most recent bone scan, CT scan and/or MRI	Secondary: rPFS, median OS	Completed	Pfizer/MDA
BMS-986249	CTLA-4	Conditio-nally activated antibody	NCT03369223, ph 1/2 (in combination with nivolumab)	Measurable disease or metastatic disease documented by bone lesions in radionuclide bone scan	Secondary: PFS, overall response, duration of response	Recruiting	CytomX Therapeutics/Bristol-Myers Squibb
Epacadostat and MVA-BN Brachyury	IDO1 TBXT	Small molecule Protein	NCT03493945, ph1/2 (in combination with M7824 and N-803)	Radiographically proven metastatic or locally advanced solid tumor of any type	Secondary: PFS	Recruiting	Incyte/NCI Bavarian Nordic
Talabostat mesylate (BXCL701)	DPP4, DPP8, DPP9, FAP	Small molecule	NCT03910660, ph1/2 (in combination with pembrolizumab)	RECIST 1.1 measurable disease or detectable bone metastases by whole body bone scintigraphy	Secondary: rPFS, median OS, duration of response	Recruiting	BioXcel Therapeutics
Dendritic cell vaccine	CTAG1B, MAGEC2, MUC1	Cell vaccine	NCT02692976, ph2	Bone disease progression defined by two or more new lesions in bone scan as described in PCWG2 criteria	Secondary: rPFS, OS	Completed	Radbound University
MB-105	PSCA	CAR-T	NCT03873805, ph1	Radiographic evidence of new metastatic foci in computed CT or bone scan	Secondary: rPFS, OS	Recruiting	Fortress Biotech/City of Hope Med Cent
Reolysin (pelareorep)	N/A	Virus	NCT01619813, ph2 (in combination with docetaxel and prednisone)	Metastatic or locally recurrent disease, clinically and/or radiologically documented disease	Primary: Disease progression, OS	Completed	Oncolytic Biotech

“Developer” refers to the original developer/owner, “Stage” refers to the highest development stage. “Inclusion” and “Outcome” columns include selected parameters relevant in the context of bone metastasis. Detailed description of the drugs and the results are provided in the chapters below. All listed trials are for prostate cancer. AR, androgen receptor; B7-H3, B7 homolog 3; CAR-T, chimeric antigen receptor, T cell; CD, cluster of differentiation; CRPC, castration-resistant prostate cancer; CTAG1B, cancer/testis antigen 1B; CTLA-4, cytotoxic T-lymphocyte-associated protein 4; DPP, dipeptidyl peptidase; FAP, fibroblast activation protein; ICAM 1, intracellular adhesion molecule 1; IL12A, interleukin 12 A; KLK3, kallikrein related peptidase 3; LBD, ligand binding domain; MAGEC2, MAGE family member C2; MRI, magnetic resonance imaging; MUC-1, mucin-1; OS, overall survival; PCWG3, prostate cancer working group 3; PD-1, programmed cell death 1; PD-L1, programmed death-ligand 1; PFS, progression-free survival; PSA, prostate-specific antigen; PSCA, prostate stem cell antigen; PSMA, prostate-specific membrane antigen; RECIST, response evaluation criteria in solid tumors; rPFS, radiographic progression-free survival; TBTX, T-box transcription factor T; TGFβ, transforming growth factor beta. N/A, Not available.

### 3.2 Imifoplatin

Imifoplatin (PT-112) is a platinum-pyrophosphate compound studied for the treatment of cancer. Due to the pyrophosphate presence, imifoplatin localizes to bone tissues in high concentration (15). Imifoplatin has been studied in patients with advanced solid tumors including bone metastatic prostate cancer (clinical trial identifier NCT02266745). The results were published in ASCO Genitourinary Cancers Symposium 2020 (16, 17), showing decrease in bone pain and reduction of serum alkaline phosphatase (ALP, bone biomarker) in 9/10 and PSA in 3/10 patients. In a combination study (NCT03409458) with avelumab in a cohort of metastatic castration-resistant prostate cancer (mCRPC), 24/32 patients showed reduction in serum ALP and improvement in patient-reported pain and quality of life (18), suggesting marked therapeutic activity for imifoplatin in bone metastasis.

### 3.3 P-PSMA-101

P-PSMA-101 is an autologous prostate-specific membrane antigen (PSMA) -targeting Chimeric Antigen Receptor T cell (CAR-T) therapy with a high percentage of stem cell memory T cell (TSCM) phenotype initially associated with efficacy, safety and bone homing. TSCM cells remain viable in hostile bone microenvironment and the bone homing attribute makes it a promising candidate treatment for bone metastasis. After promising preclinical data in prostate cancer models, a phase I clinical study (NCT04249947) was started in mCRPC patients. The results were reported in ASCO Genitourinary Symposium 2022 (19). The study included 10 heavily pretreated patients, of which 7 had decreased PSA levels. Four patients showed marked CAR-T uptake in bone metastases and post-treatment biopsy of one patient showed infiltration of P-PSMA-101 CAR-T cells into the tumor, the patient experiencing pathologic complete response. However, enrollment was stopped in the study in November 2022 when the company focused on developing their allogeneic platform.

### 3.4 Pasotuxizumab

Pasotuxizumab (BAY2010112) is a PSMA-targeting bispecific T-cell engager (BiTE) antibody. BiTE molecules bind to both the target cells and T cells, and they can recruit and activate T cells without the need of any co-stimulatory signals. Phase I dose escalation results in CRPC (NCT01723475) have been published (20). The study does not state the exact number of patients with bone metastases, but the majority of patients had advanced or metastatic stage IV disease. When PSMA BiTE was administered to patients intravenously, altogether 14/16 patients experienced a PSA response. Two patients had a long-term PSA response in the study. The first patient had a long-term stable disease, and the second patient had a marked PSA decrease and experienced a near-complete regression of lymph node lesions and bone metastases with 500 days to disease progression.

### 3.5 MGC018

MGC018 is an anti-B7-H3 antibody-drug conjugate (ADC). B7-H3, a member of the B7 family of immunomodulatory molecules, is overexpressed in a wide range of solid tumors (21). MGC018 is comprised of the cleavable linker-duocarmycin payload, valine-citrulline-*seco* duocarmycin hydroxybenzamide azaindole (vc-*seco*-DUBA), conjugated to an anti-B7-H3 humanized IgG1/kappa monoclonal antibody (22). A phase I/II study (NCT03729596) will evaluate the effects of MGC018 in patients with advanced solid tumors, including mCRPC with bone only metastases. Preliminary results of the trial presented in ESMO 2021 congress indicated that 55% of the patients experienced over 50% decrease in PSA levels (23). The study included only mCRPC patients with bone metastases, indicating a significant bone metastasis anti-tumor effect for MGC018. The effects of MGC018 are currently evaluated in advanced solid tumors, and a phase II/III trial (NCT05551117) in mCRPC patients evaluating radiographic progression free survival (rPFS) as a primary endpoint will be initiated.

B7-H3 is an interesting target for bone metastasis. A study in patients with the primary bone cancer osteosarcoma demonstrated that soluble B7-H3 levels were increased in patients with osteosarcoma compared to healthy individuals, and high levels of soluble B7-H3 correlated with tumor stage, metastases and shorter overall survival (OS) (24). Also, high levels of B7-H3 correlated with low number of CD8+ tumor-infiltrating lymphocytes (25). Importantly for bone metastasis, B7-H3 has been shown to regulate the differentiation and activity of bone-forming osteoblast cells that are overactive in prostate cancer (26, 27).

### 3.6 DS-7300a

DS-7300a is an anti-B7-H3 antibody conjugated to DXd (MAAA-1181), a novel derivate of a topoisomerase I inhibitor exatecan (DX-8951f). The first results of the ongoing phase I/II trial (NCT04145622) were presented in ASCO Genitourinary symposium in 2022. Out of 29 patients treated with 6.4 to 16 mg/kg of DS-7300, six patients experienced partial response and 15 resulted in stable disease with improvements in PSA and bone metastases (28).

### 3.7 MVI-118

MVI-118 (pTVG-AR) is a plasmid DNA vaccine encoding the ligand-binding domain of the human androgen receptor. In a phase I study (NCT02411786), 55% of metastatic castration-sensitive prostate cancer (mCSPC) patients had bone metastases and 68% of the patients were progression-free at 18 months in groups receiving the vaccine with or without granulocyte-macroghage colony-stimulating factor (GM-CSF) adjuvant (29). Patients who had immunological response with interferon gamma (IFNγ) and/or granzyme B had prolonged time to develop castration resistance. The study did not include radiographic evaluation of patients even though it included a high number of patients with bone metastases. MVI-118 is currently evaluated in prostate cancer patients in combination with pembrolizumab (NCT04090528) in a study enrolling patients with bone metastases.

### 3.8 MVI-816 (pTVG-HP)

MVI-816 (pTVG-HP) is an intradermal prostatic acid phosphatase (PAP) encoding DNA plasmid vaccine. Eighteen mCRPC patients were included in a phase II study (NCT01706458) evaluating effects of sipuleucel T alone (Arm 1) or in combination with pTVG-HP (Arm 2). The patients underwent CT/bone scan in 3 months intervals for the two years follow-up period. Patients with bone metastases were unevenly distributed between the two treatment arms (22% vs 56% in Arms 1 and 2, respectively) and there were no differences between the treatment arms in median time to radiographic progression (30). However, two patients in Arm 2 seemed to be progression free until 9 to 12 months. Overall, the number of responders was low and the study was unable to recruit enough patients, and it was closed early as it was unlikely to meet the primary immunological endpoint.

In another phase II study (NCT02499835) the effects of MVI-816 and pembrolizumab were evaluated in mCRPC patients on 6 months progression-free survival (PFS) and time to radiographic progression. In the study, 80-100% of patients had bone metastases and 32% remained on trial without radiographic progression after 6 months. Estimated rPFS rate was 44% and 62% in patients receiving a combination of MVI-816 and pembrolizumab in two dosing sequences (31). In the same study, the effects of pTVG-HP in combination with pembrolizumab were studied by FLT PET/CT imaging and different metastases were analyzed (32). Unfortunately, bone metastases were not analyzed in this study due to high background FLT uptake in PET/CT imaging from the proliferating bone marrow. If it would have been possible to image bone metastases, the study would have given important metastasis-specific information.

### 3.9 Recombinant Ad5 vaccine

A novel Ad5 vaccine uses adenovirus 5 vectors targeting tumor-associated antigens PSA, MUC-1 and brachyury. The first-in-human trial (NCT03481816) was performed in mCRPC patients that needed to have incurable disease with radiographic progression defined either by new or growing bone lesions or growing lymph node disease with increasing PSA levels (33). Seventeen patients were included in the study, of which one patient had a partial response, five had stable disease for over six months, and five patients had confirmed decline in PSA. The study did not outline what metastases the responders had, which would have been helpful for better interpretation of the results. Median PFS was 22 weeks. All patients experienced mounted T cell responses to at least one tumor-associated antigen, whereas about half of the patients mounted immune responses to all three tumor-associated antigens. Surprisingly, despite the promising anti-cancer results, almost all currently active clinical trials of the Ad5 vaccine are related to COVID-19 research.

### 3.10 Rilimogene glafolivec

Rilimogene glafolivec (PROSTVAC) is a therapeutic cancer vaccine for mCRPC patients (34). It is a combination of two viruses encoding PSA and TRICOM co-stimulatory molecules (CD80), leukocyte function associated antigen-3 (LFA-3) and intracellular adhesion molecule-1 (ICAM-1 or CD54). The patients are first given a vaccinia virus -based vector (rilimogene galvacirepvec, PROSTVAC-V) for priming and later a recombinant fowlpox virus -based vector (rilimogene glafolivec, PROSTVAC-F) for boosting the immunity. A phase III study (NCT01322490) evaluated efficacy of PROSTVAC alone and in combination with GM-CSF over placebo in mCPRC patients. About 75% of the patients had bone metastases in each cohort but there was no effect in OS, and the most common event in patients was radiographic progression and bone pain (35). Combinations with rilimogene glafolivec are currently evaluated in 7 clinical trials for different subsets of prostate cancer.

### 3.11 Bintrafusp alfa and M9241

Bintrafusp alfa (M7824) is a first-in-class bifunctional agent targeting programmed death ligand 1 (PD-L1) moiety fused with peptide linkers to ‘trap’ transforming growth factor beta (TGFβ) in the tumor microenvironment (36). In preclinical studies, bintrafusp alfa inhibited breast cancer metastasis to lungs and its effects were more profound than those of PD-L1 or TGFβ alone, and it is also an effective combination partner with chemo- and radiation therapy (37), vaccines (38) and M9241 (NHS-IL12), an immunocytokine composed of two IL-12 heterodimers fused to an antibody with high affinity to DNA (39). Phase I clinical trials in advanced solid tumors have been reported (40, 41).

As the role of TGFβ is well established in regulating growth of bone metastases (42), it would be of interest to evaluate if bintrafusp alfa affects also bone metastasis. A trial evaluating effects of binstrafusp alfa in combination with other therapies including M9241 in mCSPC and mCRPC (NCT04633252) will perform Tc99 imaging to confirm the extent of bone metastases and follow up radiographic response rates and time to radiographic progression with estimated study completion in 2023.

### 3.12 Vudalimab

Vudalimab (XmAb20717) is a bispecific antibody that engages programmed cell death 1 (PD-1) and cytotoxic T-lymphocyte antigen-4 (CTLA-4) with limited amount of published data available. Results presented in SITC 2020 concluded a preliminary clinical finding of vudalimab in advanced solid tumors (43). This study also included CRPC patients, and 2/7 patients responded to the treatment with a more than 50% decrease in PSA and no progression in bone scans. The effects of vudalimab will be studied in combination with chemo- or targeted therapy in a phase II trial in mCRPC patients (NCT05005728). In this trial, the patients will be categorized into treatment groups based on molecular characteristics of the tumors or previous treatment history. The study will evaluate effects on bone metastases by evaluating rPFS, with expected study completion in 2024.

### 3.13 Tremelimumab

Tremelimumab is a fully-human IgG2 monoclonal antibody targeting CTLA-4 and directed to treatment of advanced melanoma, prostate, breast, colorectal and renal cancer. Especially in combination with PD-1 targeting antibodies, tremelimumab has produced long-term survival benefits in advanced patients (44). Of interest to this review, tremelimumab has been studied in combination with PD-1 targeting durvalumab in bone metastatic CRPC (45, NCT03204812). rPFS was assessed with CT and Tc-99m-MDP bone scintigraphy, and bone biopsies were collected to evaluate immune cells at baseline and after 2 and 4 doses of treatment. The study reported a rPFS of 3.7 months, and 1-, 2-, and 3-year OS were 96%, 55% and 35%, respectively. Stable disease lasting at least 6 months was observed in 6/25 patients (disease control rate of 35%). Analysis of immune cell subsets in bone metastases showed no difference in T cell populations, but the number of macrophages and neutrophils was increased during the treatment period.

Effects of tremelimumab are currently evaluated in 112 clinical studies in different cancer indications, and it was approved by FDA in combination with durvalumab for unresectable hepatocellular carcinoma in October 2022. Tremelimumab in combination with durvalumab is currently studied in bone metastatic NSCLC patients (46) and metastatic urothelial carcinoma (mUC) (47). In the NSCLC study (NCT03057106), bone metastases were associated with lower OS and PFS in patients, but the treatment had no beneficial effect. The mUC study (NCT02516241) also confirmed that patients with bone metastases had lower OS and PFS, and patients with PD-L1 -high bone metastases treated with tremelimumab, durvalumab or their combination had numerically higher OS than patients with PD-L1 -low bone metastases.

### 3.14 BMS-986249

BMS-986249 is a probody composed of ipilimumab (anti-CTLA-4 antibody) linked to a proprietary masking peptide that covers the active antigen-binding site of the antibody through a protease-cleavable linker (48). BMS-986249 is currently evaluated in a phase I clinical study (NCT03369223) including patients with advanced cancer in combination with nivolumab. Results published in ESMO 2022 congress demonstrated that regardless of cancer indication, 26/39 patients treated with BMS-986249 received partial response and 16 or 38 out of 64 patients received complete or partial response, respectively, when treated with combination of BMS-986249 and nivolumab (49). The study is currently recruiting patients, and one of the cohorts will be mCRPC patients where bone metastases will be evaluated at inclusion by radionuclide bone scan. The study is expected to complete in 2024.

### 3.15 MVA-BN Brachyury and epacadostat

MVA-BN Brachyury is a recombinant vaccine under development for the treatment of patients with advanced cancers. Brachyury is a transcription factor expressed in many cancers and associated with metastatic process and chemotherapy resistance (50). The vaccine is modified from two viruses encoding a transgene for brachyury and it induces T cell responses against CEA and MUC1. QuEST1 study (NCT03493945) evaluated effects of MVA-BN Brachyury combined with M7824 (bintrafusp alfa, discussed above), M7824 and ALT-803 (IL-15 superagonist), or M7824, ALT-803 and epacadostat (discussed below) in CRPC patients who had radiologically confirmed bone metastases or PSA progression (51). Results of the QuEST1 study published in ESMO 2020 congress indicated that 4/9 asymptomatic or minimally symptomatic CRPC patients receiving the triple combination of the vaccine, bintrafusp alfa and the IL-15 superagonist sustained PSA responses and 2/4 of them had radiographic response, whereas a similar response was only observed in 1/13 patients receiving the vaccine and bintrafusp alfa (52). Furthermore, analysis of peripheral blood mononuclear cells from these two study groups showed increase in NK cells, TCR diversity and absolute lymphocyte count together with increased serum levels of granzyme B, CD27 and CD40L, indicating an established immune reaction to the vaccination (53). Also, patients who experienced a PSA response had higher numbers of CD4+ and CD8+ cells and decreased number of immunosuppressive cells such as myeloid-derived suppressor cells and monocytes, which could partially explain the observed anti-tumor effects.

Epacadostat is an indoleamine 2,3-dioxygenase (IDO) inhibitor intended to be used for treatment of cancer (54). Results of phase I/II trials for epacadostat (55) and its combination with pembrolizumab (56) in advanced solid tumors have been published. As mentioned above, the effects of epacadostat are evaluated in Arm 3 of QuEST1 trial (NCT03493945) for mCRPC patients with the inclusion criteria metastasis to bone, organs or lymph nodes, and will follow radiographic progression of the disease with expected results by the end of 2023. We have previously performed a preclinical bone metastasis study in triple-negative breast cancer where epacadostat alone or in combination with pembrolizumab had no effect on growth of bone metastases (57). However, these results do not directly translate to prostate cancer bone metastases as they may differ immunologically from breast cancer bone metastases.

### 3.16 Talabostat mesylate

Talabostat mesylate is a small-molecule inhibitor of dipeptidyl peptidases (DPPs) 4, 8 and 9, and fibroblast activation protein that activates innate immunity (58). Effects of talabostat mesylate were studied in combination with pembrolizumab in a phase II trial in mCRPC patients (NCT03910660). In this study, 40% of the patients had bone metastases, and even after a short 9-week follow-up time, 27% of the patients showed PSA reduction (59). Further results were reported in ASCO genitourinary meeting in 2022 with similar findings, demonstrating that in a mCRPC cohort that included 44% of patients with bone metastases, 23% of all mCRPC patients had complete response and 16% had partial response (60). Even though the data does not yet indicate response rates separately for bone metastatic patients, the observed 40% of patients with bone metastases will hopefully lead to discussing the issue when the full results of the phase II study are published.

### 3.17 Dendritic cell vaccine

Radboud University is developing a novel dendritic cell vaccine with subpopulations of myeloid dendritic cells (mDC) and plasmacytoid dendritic cells (pDC) targeting CTAG1B, MAGEC2 and MUC1. Results presented in ASCO Genitourinary Cancers Symposium 2018 reported rPFS data in mCRPC patients with localized, lymph node and bone metastasis positive disease (61). In this trial (NCT02692976) mCRPC patients received either mDC, pDC or combined vaccination and their radiological responses were assessed on 68GA-PSMA-PET-CT and MRI imaging for bone metastases. The results showed that overall, 13/21 patients had no radiological disease progression and at 12 months follow-up time 5/11 patients had stable disease. Mean rPFS was 6.1 months. Results published in ESMO 2019 congress reported that patients who had non-progressive disease had more antigen-specific T cells (IFNγ+) compared to progressed patients (62). rPFS was 18.8 months in patients with high IFNγ+ cells and 5.1 months in patients with low IFNγ+ cells, indicating that immune activation as seen by an elevated amount of IFNγ+ cells would mediate prolonged rPFS in patients.

### 3.18 MB-105

MB-105 is a prostate stem cell antigen (PSCA) -CAR T cell therapy currently studied in mCRPC. Prostate cancer is an immunologically cold tumor often infiltrated with abundant macrophages, and infiltration of M2 macrophages correlates with metastasis and poor prognosis. To study the effects of MB-105 at preclinical stages, prostate cancer cells were intratibially injected into ‘humanized mice’ with human immune cells to model the immunosuppressed microenvironment in a bone metastatic disease (63). PD-L1 expression was observed in tumor-associated macrophages infiltrating tumors following the PSCA-CAR T cell therapy. Importantly, treatment with anti-PD-L1 monoclonal antibodies rescued anti-tumor activity of PSCA-CAR T cells in the presence of M2 macrophages, suggesting that PD-L1 is a mediator of M2 macrophage -driven immune suppression in prostate cancer. PSCA-CAR T cell therapy is currently evaluated in a phase 1 clinical trial (NCT03873805) in mCRPC patients with PSCA-positive tumors. Results presented in ASCO Genitourinary Symposium in 2022 (64) indicated that when given with prior lymphodepletion at an optimized dose level, 3/3 patients had stable disease. More patients need to be treated before making conclusions about efficacy of the therapy.

### 3.19 Reolysin

Pelareorep (Reolysin), a naturally occurring oncolytic reovirus is developed for treatment of cancer. The reovirus infects and kills cancer cells with activated Ras pathway (65). A phase II study (NCT01656538) that included patients with bone metastases showed 7 months improved OS in patients receiving combination of pelareorep and paclitaxel compared to paclitaxel alone (66). A previous study had already indicated safety in patients with advanced solid tumors (67).

Pelareorep received Fast Track Designation from FDA in 2017 for metastatic breast cancer, and its effects are currently evaluated for other metastatic cancers. Pelareorep was studied in mCRPC (NCT01619813) by CT, bone scans and bone biomarkers to evaluate the extent and response on bone metastases (68). The results were negative, and patients receiving pelareorep, docetaxel and prednisone vs patients receiving docetaxel and prednisone had no effect on survival. A recent meta-analysis in advanced or metastatic cancer patients indicated that other oncolytic virotherapies may be more effective than pelareorep (69).

### 3.20 IMM-101

IMM-101 is a vaccine derived from heat killed mycobacterium and developed for the treatment of cancer, including prostate cancer (70). A summary of case reports of 6 prostate cancer patients treated with IMM-101 demonstrated decreased PSA levels in 3 patients with symptomatic bone metastases after starting IMM-101 treatment (71). Bone metastases remained stable, or decreased in one patient who also received zoledronic acid. In these patients the disease remained stable for 2 to 9 years. These results showed that at least some patients do respond to IMM-101 with a positive response. Current clinical development of IMM-101 seems to be directed to melanoma, pancreatic and colorectal cancers.

### 3.21 Preclinical-stage assets

Database search identified three preclinical-stage therapies showing effects in bone metastasis models, rAd.DCN, EMU-116 and DUET-02. To the best of our knowledge, no clinical studies have been posted for these therapies up to date.

University of Illinois at Chicago develops an oncolytic adenovirus-expressing decorin, rAd.DCN, for the treatment of cancer. Decorin is a natural inhibitor of TGFβ that has multiple pro-metastatic and immunomodulatory properties (72). Studies of rAd.DCN in breast cancer bone metastasis models indicated that the treatment did not prevent colonization to bones but significantly decreased tumor growth in bone (73).

EMU-116 is an orally bioavailable small-molecule CXCR4 antagonist under development for the treatment of cancer. CXCR4 has been a promising cancer target for years and it is an especially interesting anti-metastatic target as it is one of the factors involved in bone-homing of cancer cells (74). Recent preclinical data shows that EMU-116 was effective in a prostate cancer bone metastasis model when combined with docetaxel (75).

DUET-02 (CpG-STAT3ASO) is a Signal Transducer and Activator of Transcription 3 (STAT3) antisense oligonucleotide (STAT3ASO) conjugated to immunostimulatory CpG oligodeoxynucleotides that is currently being explored for the treatment of cancer. STAT3 is an oncogenic transcription factor that plays an important role in both prostate cancer progression and sustaining immune suppression in the tumor microenvironment. At preclinical stages, DUET-02 effectively prevented tumor growth and improved survival in an intratibial (tumor growing in bone) bone metastasis model (76).

## 4 Discussion

Based on the database search we identified 24 therapies in development that were evaluated in the context of bone metastasis. It is challenging to draw conclusions of which therapies could be most successful in the future, because these experimental therapies are in different stages of development, they have been tested in different patient populations, different modalities have been studied, and some of them are evaluated in combination with other therapies.

Three of the identified therapies were specifically related to tumor growing in bone metastatic microenvironment and their clinical evaluation followed outcomes in bone metastatic patients with relevant outcome measurements. These therapies included a first-in-class platinum-pyrophosphate conjugate small molecule imifoplatin (see chapter 4.2), an autologous PSMA-targeting CAR-T therapy P-PSMA-101 (chapter 4.3), and a B7-H3 targeting ADC MGC018 (chapter 4.5). Imifoplatin has affinity for bone (osteotropism) ensuring specific accumulation to bone metastases. It causes immunogenic cell death leading to recruitment of tumor-infiltrating lymphocytes (17) and it has been studied in combination with anti-PD-L1 in mCRPC patients, highlighting the rationale for the studied combination (18 ASCO). P-PSMA-101 has TSCM phenotype (19), which has been considered important for its bone marrow homing, surviving and tumor eliminating properties in mCRPC patients with bone metastases. The properties of bone homing and survival can be considered essential features for an effective therapy. However, despite of the promising phase I results, winding down was recently announced for this autologous CAR-T program and transition to an allogeneic platform. B7-H3 is a cell surface immunomodulatory glycoprotein expressed during prostate cancer progression and in the majority of patients with mCRPC (77). Interestingly, B7-H3 has been reported also to affect differentiation and activity of bone-forming osteoblast cells that are overactive in osteoblastic bone metastases typical for prostate cancer (26, 27). This indicates that targeting B7-H3 could potentially prevent the formation of pathologic osteoblastic bone lesions in mCRPC, currently an unmet medical need. Taking into account B7-H3 expression in tumor and immune cells with potential activity also in osteoblasts, B7-H3 can be considered as a potential OIO target in prostate cancer patients with bone metastases.

Some other clinical-stage therapies identified in this review have shown potential efficacy on bone metastases, including for example MVI-118, the dendritic cell vaccine and pasotuximab. However, comparison of these therapies is very difficult as the studies evaluated responders differently and used different outcomes, for example PFS or OS that do not evaluate efficacy for bone metastases as such. It is also important to acknowledge that not all therapies have shown good effects on bone metastases. For example, the study of rilimogene glafolivec addressed efficacy on bone metastases but failed to show effects on survival, bone metastasis progression or bone pain. Furthermore, different bone metastatic cancers can have differential efficacy on a therapy as shown in the case of reolysin that received fast track designation from the FDA for metastatic breast cancer but showed no efficacy on metastatic prostate cancer when both studies included patients with bone metastases. The immune microenvironment can be very different in breast and prostate cancer bone metastases, being affected by the osteolytic or osteoblastic nature, respectively, which could explain the results. These findings highlight the heterogeneity of bone metastases and the need to study effects on different cancer types separately, and not only rely on data obtained from one cancer type.

This data search resulted only in three preclinical-stage immunotherapies that have been tested in preclinical bone metastasis models. These therapies include rAD.DCN, DUET-02, and EMU-116 in combination with docetaxel that decreased bone metastasis growth in animal models. None of these experimental therapies appear to have proceeded to clinical trials yet. It will be interesting to see how the preclinical efficacy translates to clinical effectiveness on bone metastases. On the other hand, almost none of the clinical-stage therapies listed in this review have published results available on preclinical data supporting continuation to clinical studies for treatment of patients with bone metastases, with the exception of MB-105 that has reported data available from bone metastasis animal models and early data from a phase 1 clinical trial. Some other drugs had data showing effects for inhibiting or decreasing growth of lymph node or lung metastases, but these metastases are very different from bone metastases and results obtained on other metastases are usually not translatable to bone metastases. One reason for the lack of preclinical studies in bone metastasis models could be the special expertise needed to carry out studies in these technically challenging models (78). We have recently published results on how effects of immunotherapies can be addressed in proper metastasis models (79, 80) and we hope that metastasis models would be extensively used at preclinical development stages to confirm the efficacy before initiating clinical studies. This approach would lead to selecting most promising drug candidates for bone metastases to proceed to clinical trials, which would decrease the currently very high 97% failure rate in oncology clinical trials (81), and allow faster entrance of truly efficacious new oncology drugs to the market.

Considering that bone metastases are a high unmet medical need, it is surprising how few relevant studies finally address the efficacy of novel therapies on bone metastases either in preclinical or clinical studies. In fact, the efficacy of novel therapies on bone metastases should be specifically addressed as bone metastases are associated with very low response rates to therapies and shorter PFS and OS rates (46). For this reason, it has been proposed that bone metastases should be considered as a new important stratification factor for clinical trials evaluating effects of immunotherapies (46). Guidance on how to study and evaluate effects of therapies on bone metastases in clinical trials is not well established. The RECIST criteria often advise to exclude bone metastases in monitoring the response of experimental therapies. PCWG3 criteria, that are often used to evaluate responses in prostate cancer, advise bone imaging as part of evaluating effects on bone metastases, but states that more data is needed to understand the data collected by imaging tools in evaluating responses on bone metastases. In fact, results of bone scans can be sometimes misleading. New bone scan lesions may represent osteoblastic bone healing defined as bone pseudoprogression and mistakenly diagnosed as disease progression (82). Bone-related biomarkers have been developed and used in bone-related diseases for a long time, but they remain mainly unexplored with bone metastases (83). In our opinion, bone turnover markers should be more widely studied and used in clinical trials for early detection of bone metastases and evaluating efficacy of therapies. Furthermore, there are some promising developments for bone metastasis -specific biomarkers such as DKK-1 (84). The use of hormone-deprivation therapies, either anti-estrogens or anti-androgens, induces bone changes in patients, further complicating the analysis of bone metastasis results. These and other complexities in the follow-up together with the limited guidance are probably among the main reasons why there are so few studies evaluating specific effects on bone metastases. In this review we identified concrete evidence on these issues. The initial data search was performed for breast and prostate cancer, but a more detailed evaluation of the data showed that only clinical trials in prostate cancer studied effects on bone metastases. Besides breast and prostate cancer, many other common cancers such as lung, colon and bladder cancer and melanoma have a high incidence of bone metastases, and bone metastasis -specific evaluation should be applied to all these cancer indications in studies that include bone metastatic patients.

This review provides important timely insights to new and emerging immunotherapies with evidence for effects on bone metastases. Publication strategies of drug development companies heavily depend on intellectual property right issues. Therefore, studies are often published during later development phases and data sources are important tools to follow real-time drug development. Most of the data that was used for preparing this manuscript was obtained from non-peer-reviewed sources such as meeting abstracts, and readers should consider this when making interpretations of the data presented. We previously performed a search for clinical bone metastasis studies and immuno-oncology drug development publications and concluded that the number of peer-reviewed publications in this area is very low (85). However, considering importance of this topic and knowing there are opportunities in development, we wanted to perform an expanded data search using a database with a tailor-made filter for finding therapies with data available for bone metastasis from more abundant information sources such as news, patents and meeting abstracts that include latest published data available.

In summary, this review provides insights to novel immuno-oncology drug development for bone metastasis. Because OIO is still a largely unexplored area, conducting clinical trials in bone metastasis setting is challenging. Publication practices during drug development provide their own challenges for obtaining information, but we were able to identify novel therapies with targets or properties relevant to bone metastasis with promising data obtained during early-stage development. According to this review, bone metastases can be approached using different modalities and the tumor microenvironment in bone provides many potential targets in immune, bone and tumor cells. In the future, we will hopefully see more therapies with bone metastasis specific targets that have provided both preclinical and clinical proof-of-concept for efficacy on bone metastases.

## Author contributions

TK: writing the first version of the manuscript, manuscript modification and finalization. GM: adaptation of the database machine learning algorithms to identify associations and relationships to bone metastasis within IO drug trials. RA: subject expertise on the database, commenting and confirming that statements related to the database are correctly presented. JH: commenting and modifying the manuscript, language corrections. JB: concept for the manuscript, commenting and confirming that the data is properly presented. All authors contributed to the article and approved the submitted version.

## References

[1] Coleman RE Croucher PI Padhani AR Clézardin P Chow E Fallon M. Bone metastases. Nat Rev Dis Primers (2020) 6(1):83. doi: 10.1038/s41572-020-00216-3 33060614

[2] Svensson E Christiansen CF Ulrichsen SP Rørth MR Sørensen HT. Survival after bone metastasis by primary cancer type: a Danish population-based cohort study. BMJ Open (2017) 7(9):e016022. doi: 10.1136/bmjopen-2017-016022 PMC5595184 28893744

[3] Yang M Liu C Yu X. Skeletal-related adverse events during bone metastasis of breast cancer: current status. Discovery Med (2019) 27(149):211–20. 31361984

[4] Liu C Wang M Xu C Li B Chen J Chen J. Immune checkpoint inhibitor therapy for bone metastases: specific microenvironment and current situation. J Immunol Res (2021) 2021:8970173. doi: 10.1155/2021/8970173 34877360 PMC8645368

[5] Kfoury Y Baryawno N Severe N Mei S Gustafsson K Hirz T. Human prostate cancer bone metastases have an actionable immunosuppressive microenvironment. Cancer Cell (2021) 39(11):1464–1478.e8. doi: 10.1016/j.ccell.2021.09.005 34719426 PMC8578470

[6] Xiang L Gilkes DM. The contribution of the immune system in bone metastasis pathogenesis. Int J Mol Sci (2019) 20(4):999. doi: 10.3390/ijms20040999 30823602 PMC6412551

[7] Tahara RK Brewer TM Theriault RL Ueno NT. Bone metastasis of breast cancer. Adv Exp Med Biol (2019) 1152:105–29. doi: 10.1007/978-3-030-20301-6_7 31456182

[8] Owen KL Parker BS. Beyond the vicious cycle: the role of innate osteoimmunity, automimicry and tumor-inherent changes in dictating bone metastasis. Mol Immunol (2019) 110:57–68. doi: 10.1016/j.molimm.2017.11.023 29191489

[9] Li H Hong S Qian J Zheng Y Yang J Yi Q. Cross talk between the bone and immune systems: osteoclasts function as antigen-presenting cells and activate CD4+ and CD8+ T cells. Blood (2010) 116(2):210–7. doi: 10.1182/blood-2009-11-255026 PMC2910608 20304810

[10] Monteiro AC Bonomo A. CD8+ T cells from experimental *in situ* breast carcinoma interfere with bone homeostasis. Bone (2021) 150:116014. doi: 10.1016/j.bone.2021.116014 34022456

[11] Kähkönen TE Halleen JM Bernoulli J. Osteoimmuno-oncology: therapeutic opportunities for targeting immune cells in bone metastasis. Cells (2021) 10(6):1529. doi: 10.3390/cells10061529 34204474 PMC8233913

[12] Moseley KF Naidoo J Bingham CO Carducci MA Forde PM Gibney GT. Immune-related adverse events with immune checkpoint inhibitors affecting the skeleton: a seminal case series. J Immunother Cancer (2018) 6(1):104. doi: 10.1186/s40425-018-0417-8 30305172 PMC6180387

[13] Angelopoulou F Bogdanos D Dimitroulas T Sakkas L Daoussis D. Immune checkpoint inhibitor-induced musculoskeletal manifestations. Rheumatol Int (2021) 41(1):33–42. doi: 10.1007/s00296-020-04665-7 32743706

[14] Hofbauer LC Bozec A Rauner M Jakob F Perner S Pantel K. Novel approaches to target the microenvironment of bone metastasis. Nat Rev Clin Oncol (2021) 18(8):488–505. doi: 10.1038/s41571-021-00499-9 33875860

[15] Ames TD Sharik ME Rather GM Hochart G Bonnel D Linehan S. Translational research of PT-112, a clinical agent in advanced phase I development: evident bone tropism, synergy *In vitro* with bortezomib and lenalidomide , and potent efficacy in the Vk*MYC mouse model of multiple myeloma. Blood (2017) 130(Supplement 1):1797.

[16] Bryce AH Dronca RS Costello BA Infante JR Ames TD Jimeno J. PT-112 in advanced metastatic castrate-resistant prostate cancer (mCRPC), as monotherapy or in combination with PD-L1 inhibitor avelumab: findings from two phase I studies. J Clin Oncol (2020) 38(suppl 6; abstr 83). doi: 10.1200/JCO.2020.38.6_suppl.83

[17] Karp DD Camidge DR Infante JR Ames TD Price MR Jimeno J. Phase I study of PT-112, a novel pyrophosphate-platinum immunogenic cell death inducer, in advanced solid tumours. EClinicalMedicine (2022) 49:101430. doi: 10.1016/j.eclinm.2022.101430 35747193 PMC9156977

[18] Bryce AH Dronca RS Costello BA Aparicio A Subudhi SK O'Donnell JF. A phase 1b study of novel immunogenic cell death inducer PT-112 plus PD-L1 inhibitor avelumab in metastatic castrate-resistant prostate cancer (mCRPC) patients. J Clin Oncol (2021) 39(suppl 15; abstr e17025). doi: 10.1200/JCO.2021.39.15_suppl.e17025

[19] Slovin SF Dorff TB Falchook GS Wei XX Gao X McKay RR. Phase 1 study of p-PSMA-101 CAR-T cells in patients with metastatic castration-resistant prostate cancer (mCRPC). J Clin Oncol (2022) 40(suppl 6; abstr 98). doi: 10.1200/JCO.2022.40.6_suppl.098

[20] Hummel HD Kufer P Grüllich C Seggewiss-Bernhardt R Deschler-Baier B Chatterjee M. Pasotuxizumab, a BiTE® immune therapy for castration-resistant prostate cancer: phase I, dose-escalation study findings. Immunotherapy (2021) 13(2):125–41. doi: 10.2217/imt-2020-0256 33172323

[21] Scribner JA Brown JG Son T Chiechi M Li P Sharma S. Preclinical development of MGC018, a duocarmycin-based antibody-drug conjugate targeting B7-H3 for solid cancer. Mol Cancer Ther (2020) 19(11):2235–44. doi: 10.1158/1535-7163.MCT-20-0116 32967924

[22] Scribner JA Chiechi M Li P Son T Hooley J Li Y. MGC018, a duocarmycin-based antibody-drug conjugate targeting B7-H3, exhibits immunomodulatory activity and enhanced antitumor activity in combination with checkpoint inhibitors. Cancer Research (2020) 80(16, Suppl):Abstract 5203.

[23] Shenderov E Mallesara GHG Wysocki PJ Xu W Ramlau R Weickhardt AJ. 620P - MGC018, an anti-B7-H3 antibody-drug conjugate (ADC), in patients with advanced solid tumors: preliminary results of phase I cohort expansion. Ann Oncol (2021) 32(suppl_5):S626–77. doi: 10.1016/annonc/annonc702

[24] Wang L Kang FB Zhang GC Wang J Xie MF Zhang YZ. Clinical significance of serum soluble B7-H3 in patients with osteosarcoma. Cancer Cell Int (2018) 18:115. doi: 10.1186/s12935-018-0614-z 30123093 PMC6090643

[25] Wang L Zhang Q Chen W Shan B Ding Y Zhang G. B7-H3 is overexpressed in patients suffering osteosarcoma and associated with tumor aggressiveness and metastasis. PloS One (2013) 8(8):e70689. doi: 10.1371/journal.pone.0070689 23940627 PMC3734259

[26] Suh WK Wang SX Jheon AH Moreno L Yoshinaga SK Ganss B. The immune regulatory protein B7-H3 promotes osteoblast differentiation and bone mineralization. Proc Natl Acad Sci U S A (2004) 101(35):12969–73. doi: 10.1073/pnas.0405259101 PMC516502 15317945

[27] Xu L Zhang G Zhou Y Chen Y Xu W Wu S. Stimulation of B7-H3 (CD276) directs the differentiation of human marrow stromal cells to osteoblasts. Immunobiology (2011) 216(12):1311–7. doi: 10.1016/j.imbio.2011.05.013 21893365

[28] Patel MR Johnson ML Falchook GS Doi T Friedman CF Piha-Paul SA. DS-7300 (B7-H3 DXd-ADC) in patients (pts) with metastatic castration-resistant prostate cancer (mCRPC): a subgroup analysis of a phase 1/2 multicenter study. J Clin Oncol (2022) 40(suppl 6; abstr 87). doi: 10.1200/JCO.2022.40.6_suppl.087

[29] Kyriakopoulos CE Eickhoff JC Ferrari AC Schweizer MT Wargowski E Olson BM. Multicenter phase I trial of a DNA vaccine encoding the androgen receptor ligand-binding domain (pTVG-AR, MVI-118) in patients with metastatic prostate cancer. Clin Cancer Res (2020) 26(19):5162–71. doi: 10.1158/1078-0432.CCR-20-0945 PMC7541575 32513836

[30] Wargowski E Johnson LE Eickhoff JC Delmastro L Staab MJ Liu G. Prime-boost vaccination targeting prostatic acid phosphatase (PAP) in patients with metastatic castration-resistant prostate cancer (mCRPC) using sipuleucel-T and a DNA vaccine. J Immunother Cancer (2018) 6(1):21. doi: 10.1186/s40425-018-0333-y 29534736 PMC5850960

[31] McNeel DG Eickhoff JC Wargowski E Johnson LE Kyriakopoulos CE Emamekhoo H. Phase 2 trial of T-cell activation using MVI-816 and pembrolizumab in patients with metastatic, castration-resistant prostate cancer (mCRPC). J Immunother Cancer (2022) 10(3):e004198. doi: 10.1136/jitc-2021-004198 35277461 PMC8919462

[32] Scarpelli M Zahm C Perlman S McNeel DG Jeraj R Liu G. FLT PET/CT imaging of metastatic prostate cancer patients treated with pTVG-HP DNA vaccine and pembrolizumab. J Immunother Cancer (2019) 7(1):23. doi: 10.1186/s40425-019-0516-1 30700328 PMC6354338

[33] Bilusic M McMahon S Madan RA Karzai F Tsai YT Donahue RN. Phase I study of a multitargeted recombinant Ad5 PSA/MUC-1/brachyury-based immunotherapy vaccine in patients with metastatic castration-resistant prostate cancer (mCRPC). J Immunother Cancer (2021) 9(3):e002374. doi: 10.1136/jitc-2021-002374 33762322 PMC7993215

[34] Lasek W Zapała Ł. Therapeutic metastatic prostate cancer vaccines: lessons learnt from urologic oncology. Cent Eur J Urol (2021) 74(3):300–7. doi: 10.5173/ceju.2021.0094 PMC8552937 34729217

[35] Gulley JL Borre M Vogelzang NJ Ng S Agarwal N Parker S. Phase III trial of PROSTVAC in asymptomatic or minimally symptomatic metastatic castration-resistant prostate cancer. J Clin Oncol (2019) 37:1051–61. doi: 10.1200/JCO.18.02031 PMC6494360 30817251

[36] Gameiro SR Strauss J Gulley JL Schlom J. Preclinical and clinical studies of bintrafusp alfa, a novel bifunctional anti-PD-L1/TGFβRII agent: current status. Exp Biol Med (Maywood) (2022) 247(13):1124–34. doi: 10.1177/15353702221089910 PMC9335510 35473390

[37] Lan Y Zhang D Xu C Hance KW Marelli B Qi J. Enhanced preclinical antitumor activity of M7824, a bifunctional fusion protein simultaneously targeting PD-L1 and TGF-β. Sci Transl Med (2018) 10(424):eaan5488. doi: 10.1126/scitranslmed.aan5488 29343622

[38] Knudson KM Hicks KC Luo X Chen JQ Schlom J Gameiro SR. M7824, a novel bifunctional anti-PD-L1/TGFβ trap fusion protein, promotes anti-tumor efficacy as monotherapy and in combination with vaccine. Oncoimmunology (2018) 7(5):e1426519. doi: 10.1080/2162402X.2018.1426519 29721396 PMC5927523

[39] Xu C Marelli B Qi J Qin G Yu H Wang H. NHS-IL12 and bintrafusp alfa combination therapy enhances antitumor activity in preclinical cancer models. Transl Oncol (2022) 16:101322. doi: 10.1016/j.tranon.2021.101322 34954456 PMC8718653

[40] Strauss J Heery CR Schlom J Madan RA Cao L Kang Z. Phase I trial of M7824 (MSB0011359C), a bifunctional fusion protein targeting PD-L1 and TGFβ, in advanced solid tumors. Clin Cancer Res (2018) 24(6):1287–95. doi: 10.1158/1078-0432.CCR-17-2653 PMC7985967 29298798

[41] Doi T Fujiwara Y Koyama T Ikeda M Helwig C Watanabe M. Phase I study of the bifunctional fusion protein bintrafusp Alfa in Asian patients with advanced solid tumors, including a hepatocellular carcinoma safety-assessment cohort. Oncologist (2020) 25(9):e1292–302. doi: 10.1634/theoncologist.2020-0249 PMC7485354 32324927

[42] Juárez P Guise TA. TGF-β in cancer and bone: implications for treatment of bone metastases. Bone (2011) 48(1):23–9. doi: 10.1016/j.bone.2010.08.004 20699127

[43] Shum E Daud A Reilley M Najjar Y Thompson J Baranda J. Preliminary safety, pharmacokinetics/pharmacodynamics, and antitumor activity of XmAb20717, a PD-1 x CTLA-4 bispecific antibody, in patients with advanced solid tumors. JITC (2020) 8(3):A247–8. doi: 10.1136/jitc-2020-SITC2020.0407

[44] Comin-Anduix B Escuin-Ordinas H Ibarrondo FJ. Tremelimumab: research and clinical development. Onco Targets Ther (2016) 9:1767–76. doi: 10.2147/OTT.S65802 PMC4809326 27042127

[45] Subudhi SK Siddiqui BA Aparicio AM Yadav SS Basu S Chen H. Combined CTLA-4 and PD-L1 blockade in patients with chemotherapy-naïve metastatic castration-resistant prostate cancer is associated with increased myeloid and neutrophil immune subsets in the bone microenvironment. J Immunother Cancer (2021) 9(10):e002919. doi: 10.1136/jitc-2021-002919 34663638 PMC8524287

[46] Leitzel K Ali AM Ding K Leighl NB Badillo FEV Gaudreau P-O. Treatment with or without chemotherapy in high-risk, stage IVA/B NSCLC. J Clin Oncol (2022) 40(16_suppl):9067–7. doi: 10.1200/JCO.2022.40.16_suppl.9067

[47] Stecca C Abdeljalil O Lu C Zhang H Goluboff ET Sridhar SS. Prognostic impact of bone metastasis in patients with metastatic urothelial carcinoma (mUC) treated with durvalumab (D) with or without tremelimumab (T) in the DANUBE study. J Clin Oncol (2022) 40(16_suppl):4564–4. doi: 10.1200/JCO.2022.40.16_suppl.4564 39353213

[48] Engelhardt J Akter R Loffredo J Bezman N So P Tipton K. Preclinical characterization of novel anti-CTLA-4 prodrug antibodies with an enhanced therapeutic index. Cancer Research (2020) 80(16, Suppl):Abstract 4551.

[49] Gutierrez M Friedman CF Long GV Ascierto PA Melero I Richards D. 740P - anti-cytotoxic T-lymphocyte antigen-4 (CTLA 4) probody BMS-986249 ± nivolumab (NIVO) in patients (pts) with advanced cancers: updated phase I results. Ann Oncol (2022) 33(suppl_7):S331–55. doi: 10.1016/annonc/annonc1058

[50] Collins JM Donahue RN Tsai YT Manu M Palena C Gatti-Mays ME. Phase I trial of a modified vaccinia Ankara priming vaccine followed by a fowlpox virus boosting vaccine modified to express brachyury and costimulatory molecules in advanced solid tumors. Oncologist (2020) 25(7):560–e1006. doi: 10.1634/theoncologist.2019-0932 31876334 PMC7356719

[51] Redman JM Steinberg SM Gulley JL. Quick efficacy seeking trial (QuEST1): a novel combination immunotherapy study designed for rapid clinical signal assessment metastatic castration-resistant prostate cancer. J Immunother Cancer (2018) 6(1):91. doi: 10.1186/s40425-018-0409-8 30227893 PMC6145343

[52] Redman JM Madan RA Karzai F Bilusic M Cordes L Marte J. Efficacy of BN-brachyury (BNVax) + bintrafusp alfa (BA) + N-803 in castration-resistant prostate cancer (CRPC): Results from a preliminary analysis of the Quick Efficacy Seeking Trial (QuEST1). Annals of Oncol (2020) 31(Suppl 4):Abstract 616MO.

[53] Toney N Tsai Y Redman J Gulley J Schlom J Donahue R. 582 immune correlates from QuEST1 in men with castration-resistant prostate cancer. J ImmunoTherapy Cancer (2021) 9. doi: 10.1136/jitc-2021-SITC2021.582

[54] Prendergast GC Malachowski WJ Mondal A Scherle P Muller AJ. Indoleamine 2,3-dioxygenase and its therapeutic inhibition in cancer. Int Rev Cell Mol Biol (2018) 336:175–203. doi: 10.1016/bs.ircmb.2017.07.004 29413890 PMC6054468

[55] Beatty GL O'Dwyer PJ Clark J Shi JG Bowman KJ Scherle PA. First-in-Human phase I study of the oral inhibitor of indoleamine 2,3-Dioxygenase-1 epacadostat (INCB024360) in patients with advanced solid malignancies. Clin Cancer Res (2017) 23(13):3269–76. doi: 10.1158/1078-0432.CCR-16-2272 PMC5496788 28053021

[56] Mitchell TC Hamid O Smith DC Bauer TM Wasser JS Olszanski AJ. Epacadostat plus pembrolizumab in patients with advanced solid tumors: phase I results from a multicenter, open-label phase I/II trial (ECHO-202/KEYNOTE-037). J Clin Oncol (2018) 36(32):3223–30. doi: 10.1200/JCO.2018.78.9602 PMC6225502 30265610

[57] Kähkönen TE Suominen MI Mäki-Jouppila JH Halleen JM Bernoulli J. Efficacy of anti-PD-1, IDO inhibitor, chemotherapy and bone-targeting agent on tumor growth in a syngeneic bone metastasis model of triple-negative breast cancer. Cancer Res (2020) 80(16, Suppl):Abstract 5026.

[58] Cunningham CC. Talabostat. Expert Opin Investig Drugs (2007) 16(9):1459–65. doi: 10.1517/13543784.16.9.1459 17714031

[59] Monk P Zhang J Costin D Petrylak DP Tagawa ST Karsh LI. BXCL701 - 1st-in-class oral activator of systemic innate immunity-combined with pembrolizumab, in men with metastatic castration-resistant prostate cancer (mCRPC): phase II results. Ann Oncol (2021) 32(suppl_5):S626–77. doi: 10.1016/annonc/annonc702

[60] Zhang Y Liu C Wang T Kong F Zhang H Yi J. Therapeutic effects of mesenchymal stem cells loaded with oncolytic adenovirus carrying decorin on a breast cancer lung metastatic mouse model. Mol Ther Oncolytics (2022) 24:486–96. doi: 10.1016/j.omto.2022.01.007 PMC8850566 35229027

[61] Westdorp H van Oort IM Creemers JHA Schreibelt G Mehra N de Goede AL. Myeloid and plasmacytoid dendritic cell vaccinations for castration-resistant prostate cancer patients. J Clin Oncol (2018) 36(suppl 6S; abstr 219). doi: 10.1200/JCO.2018.36.6_suppl.219

[62] Creemers J Westdorp H van Oort I Schreibelt G Gorris M Mehra N. 1523 - natural dendritic cell vaccinations generate immune responses that correlate with clinical outcome in patients with chemo-naive castration-resistant prostate cancer. Ann Oncol (2019) 30(suppl_5):v475–532. doi: 10.1093/annonc/mdz253 PMC6854814 31727154

[63] Yamaguchi Y Gibson J Ou K Priceman S. M2 macrophage-mediated immune suppression of chimeric antigen receptor T cells *via* PD-L1 signaling in prostate cancer. J Immunother Cancer (2021) 9(Suppl 2):Abstract 224. doi: 10.1136/jitc-2021-SITC2021.224

[64] Dorff TB Blanchard S Martirosyan H Adkins L Dhapola G Moriarty A. Phase 1 study of PSCA-targeted chimeric antigen receptor (CAR) T cell therapy for metastatic castration-resistant prostate cancer (mCRPC). J Clin Oncol (2022) 40(suppl 6; abstr 91). doi: 10.1200/JCO.2022.40.6_suppl.091

[65] Stoeckel J Hay JG. Drug evaluation: reolysin–wild-type reovirus as a cancer therapeutic. Curr Opin Mol Ther (2006) 8(3):249–60. 16774045

[66] Bernstein V Ellard SL Dent SF Tu D Mates M Dhesy-Thind SK. A randomized phase II study of weekly paclitaxel with or without pelareorep in patients with metastatic breast cancer: final analysis of Canadian cancer trials group IND.213. Breast Cancer Res Treat (2018) 167(2):485–93. doi: 10.1007/s10549-017-4538-4 29027598

[67] Gollamudi R Ghalib MH Desai KK Chaudhary I Wong B Einstein M. Intravenous administration of reolysin, a live replication competent RNA virus is safe in patients with advanced solid tumors. Invest New Drugs (2010) 28(5):641–9. doi: 10.1007/s10637-009-9279-8 PMC3851036 19572105

[68] Eigl BJ Chi K Tu D Hotte SJ Winquist E Booth CM. A randomized phase II study of pelareorep and docetaxel or docetaxel alone in men with metastatic castration resistant prostate cancer: CCTG study IND 209. Oncotarget (2018) 9(8):8155–64. doi: 10.18632/oncotarget.24263 PMC5814290 29487723

[69] Xie R Bi X Shang B Zhou A Shi H Shou J. Efficacy and safety of oncolytic viruses in advanced or metastatic cancer: a network meta-analysis. Virol J (2021) 18(1):158. doi: 10.1186/s12985-021-01630-z 34332591 PMC8325792

[70] Stavrinides V Dalgleish A Copier JP Moore CM. Mycobacterial immunotherapy for prostate cancer: where can we go from here? Nat Rev Urol (2020) 17(4):189–90. doi: 10.1038/s41585-020-0283-2 31996816

[71] Dalgleish AG Liu WM. The role of immune modulation and anti-inflammatory agents in the management of prostate cancer: a case report of six patients. Oncol Lett (2022) 24(2):247. doi: 10.3892/ol.2022.13367 35761946 PMC9214706

[72] Zhang J Aggarwal RR Tagawa ST Linch MD Petrylak DP Costin D. BXCL701: first-in-class oral activator of systemic innate immunity combined with pembrolizumab, in patients with metastatic castration-resistant prostate cancer (mCRPC) of adenocarcinoma phenotype–phase 2a results. J Clin Oncol (2022) 40(6_suppl):125–5. doi: 10.1200/JCO.2022.40.6_suppl.125

[73] Yang Y Xu W Neill T Hu Z Wang CH Xiao X. Systemic delivery of an oncolytic adenovirus expressing decorin for the treatment of breast cancer bone metastases. Hum Gene Ther (2015) 26(12):813–25. doi: 10.1089/hum.2015.098 PMC4692115 26467629

[74] Chatterjee S Behnam Azad B Nimmagadda S. The intricate role of CXCR4 in cancer. Adv Cancer Res (2014) 124:31–82. doi: 10.1016/B978-0-12-411638-2.00002-1 25287686 PMC4322894

[75] Miller EJ Sun CQ Gregorova P Jecs E Tahirovic YA Wilson RJ. Orally bioavailable small molecule CXCR4 antagonists with enhanced efficacy in mouse models of genitourinary cancers. Cancer Res (2022) 82(12, Suppl):Abstract 2649.

[76] Moreira D Adamus T Zhao X Su YL Zhang Z White SV. STAT3 inhibition combined with CpG immunostimulation activates antitumor immunity to eradicate genetically distinct castration-resistant prostate cancers. Clin Cancer Res (2018) 24(23):5948–62. doi: 10.1158/1078-0432.CCR-18-1277 PMC6279477 30337279

[77] Guo C Figueiredo I Gurel B Neeb A Seed G Crespo M. B7-H3 as a therapeutic target in advanced prostate cancer. Eur Urol (2023) 83(3):224–38. doi: 10.1016/j.eururo.2022.09.004 PMC7617982 36114082

[78] Kähkönen TE Bernoulli J Halleen JM Suomienen M. Novel and conventional preclinical models to investigate bone metastasis. Curr Mol Bio Rep (2019) 5:48–54. doi: 10.1007/s40610-019-0114-5

[79] Kähkönen TE Halleen JM Bernoulli J. Immunotherapies and metastatic cancers: understanding utility and predictivity of human immune cell engrafted mice in preclinical drug development. Cancers (Basel) (2020) 12(6):1615. doi: 10.3390/cancers12061615 32570871 PMC7352707

[80] Kähkönen TE Halleen JM Bernoulli J. Preclinical osteoimmuno-oncology models to study effects of immunotherapies on bone metastasis. In: Animal Models for the Development of Cancer Immunotherapy. Hoboken (NJ), USA: John Wiley & Sons, Inc (2022). p. 167–209.

[81] Wong CH Siah KW Lo AW. Estimation of clinical trial success rates and related parameters. Biostatistics (2019) 20(2):273–86. doi: 10.1093/biostatistics/kxx069 PMC6409418 29394327

[82] Lin M Jin Y Yang Z Hu X Zhang J. Determination and clinical significance of bone pseudoprogression in hormone receptor-positive metastatic breast cancer. Ther Adv Med Oncol (2021) 13:17588359211022881. doi: 10.1177/17588359211022881 34188696 PMC8209838

[83] Yamamichi G Kato T Yumiba S Tomiyama E Koh Y Nakano K. Diagnostic and prognostic significance of tartrate-resistant acid phosphatase type 5b in newly diagnosed prostate cancer with bone metastasis: a real-world multi-institutional study. Int J Urol (2022) 28. doi: 10.1111/iju.15063 PMC10092858 36305578

[84] Shi T Liang K Zhou X Song X Wang H Zhang Y. 1382 DKK1 is a biomarker and immunotherapeutic target for bone metastases in malignant cancers. J ImmunoTherapy Cancer (2022) 10. doi: 10.1136/jitc-2022-SITC2022.1382

[85] Kähkönen TE Halleen JM Bernoulli J. Limited data from clinical trials assessing immunotherapy effects on bone metastases. Cancer Res (2021) 81(13, Suppl):Abstract 2870.

